# Catalysis and Structure of Zebrafish Urate Oxidase Provide Insights into the Origin of Hyperuricemia in Hominoids

**DOI:** 10.1038/srep38302

**Published:** 2016-12-06

**Authors:** Marialaura Marchetti, Anastasia Liuzzi, Beatrice Fermi, Romina Corsini, Claudia Folli, Valentina Speranzini, Francesco Gandolfi, Stefano Bettati, Luca Ronda, Laura Cendron, Rodolfo Berni, Giuseppe Zanotti, Riccardo Percudani

**Affiliations:** 1 Department of Life Sciences, University of Parma, 43124, Parma, Italy; 2 Department of Food Science University of Parma, 43124, Parma, Italy; 3 Department of Neurosciences, University of Parma, 43124, Parma, Italy; 4 Department of Biology, University of Padova, 35121, Padova, Italy

## Abstract

Urate oxidase (Uox) catalyses the first reaction of oxidative uricolysis, a three-step enzymatic pathway that allows some animals to eliminate purine nitrogen through a water-soluble compound. Inactivation of the pathway in hominoids leads to elevated levels of sparingly soluble urate and puts humans at risk of hyperuricemia and gout. The uricolytic activities lost during evolution can be replaced by enzyme therapy. Here we report on the functional and structural characterization of Uox from zebrafish and the effects on the enzyme of the missense mutation (F216S) that preceded Uox pseudogenization in hominoids. Using a kinetic assay based on the enzymatic suppression of the spectroscopic interference of the Uox reaction product, we found that the F216S mutant has the same turnover number of the wild-type enzyme but a much-reduced affinity for the urate substrate and xanthine inhibitor. Our results indicate that the last functioning Uox in hominoid evolution had an increased Michaelis constant, possibly near to upper end of the normal range of urate in the human serum (~300 μM). Changes in the renal handling of urate during primate evolution can explain the genetic modification of uricolytic activities in the hominoid lineage without the need of assuming fixation of deleterious mutations.

Urate oxidase (uricase, Uox) catalyses hydroxylation of urate to give 5-hydroxyisourate (HIU)^1^. This is the first and rate-limiting step of uricolysis, a three-step enzymatic pathway present in both prokaryotes and eukaryotes that converts urate into the more soluble (*S*)-allantoin^2^. Uox is an enzyme of medical and evolutionary importance. Its loss by pseudogenization during primate evolution^3^,^4^, caused the inactivation of the uricolytic pathway in hominoids (lesser and great apes, including humans). The inability to degrade urate coupled with an effective renal reabsorption system for this compound leads to elevated concentration of urate in blood^5^,^6^, arguably the most noticeable difference in hematological parameters between hominoids and other mammals. Excess of serum urate (hyperuricemia) is commonly defined in humans for concentrations exceeding 400 μM; however, the normal concentrations of urate in the human serum (160–360 μM) are about ten times higher than in mammals possessing functional Uox^7^,^8^,^9^. The metabolic differences in purine degradation between hominoids and other mammals are illustrated by the consequences of deficiency of hypoxanthine-guanine phosphoribosyltransferase (HPRT), an enzyme that recycles free purines into the corresponding nucleotides. Inborn deficiency of this enzyme in humans due to mutation in the *HPRT1* gene causes early-onset severe hyperuricemia and a constellation of neurological symptoms that characterize the Lesch-Nyhan syndrome^10^,^11^. Conversely, in the mouse, complete absence of the HPRT enzyme due to experimental gene inactivation does not cause hyperuricemia and is virtually asymptomatic^12^. In humans, the intravenous administration of urate oxidase, replacing the Uox activity lost in hominoid evolution, is used in the enzymatic therapy of severe hyperuricemia and to prevent the burst of uric acid accompanying tumor lysis after certain chemotherapy treatments^13^.

Mammals able to degrade urate possess the classical type of urate oxidase (EC 1.7.3.3). The oldest known structure of the enzyme is a tetramer of the tunnelling (T) fold domain able to catalyse urate oxidation without the help of cofactors^14^,^15^. Complete bacterial genomes have revealed a surprising variety of proteins with independent origin involved in urate oxidation. Besides the cofactorless Uox, the prevalent form in Gram-positive bacteria, there are two distinct flavoenzymes^16^,^17^ and an integral membrane cytochrome *c*-containing protein, which is the prevalent Uox in Gram-negative bacteria^18^. Plants, fungi and animals possess the same cofactorless Uox, suggesting an ancient origin for this protein in eukaryote evolution.

In animals, uricolysis occurs in peroxisomes by the consecutive action of Uox, HIU hydrolase (Urah), and 2-oxo-4-hydroxy-4-carboxy-5-ureidoimidazoline (OHCU) decarboxylase (Urad). Genes orthologous to characterized Uox, Urah and Urad, are found in basal metazoan lineages such as cnidarians, consistent with the presence of the uricolytic pathway in the last common ancestor of metazoa. These genes have survived one billion years of animal evolution before being inactivated in hominoids. Although the human genome is littered with thousands of pseudogenes^19^, non-duplicated pseudogenes such as Uox are rare (<1%). At variance with the inactivation of copies of functional genes deriving from segmental duplications or retroposition, the loss of “unitary” or “long-established” genes^20^,^21^ results in the loss of a specific function in the organism. This is a rare event in mammalian genomes that is likely to be driven by adaptive evolution^22^. Genome-wide surveys have identified a very limited number of unitary gene losses (six or seven genes, including Uox) in the hominoid lineage^20^,^21^. Loss of the Uox activity determined dismissal and pseudogenization of the two other genes involved in urate degradation, Urah and Urad^23^, providing the only known example of inactivation of a metabolic pathway in the hominoid lineage.

The causes and mechanisms of Uox pseudogenization have been intensively investigated. The pseudo-coding sequence of the Uox gene in extant hominoids contains multiple stop codons. However, the lack of a common nonsense mutation in the sequence of great and lesser apes led to the proposal that the gene was independently inactivated in the two lineages^3^,^4^. More recently, it has been suggested that Uox inactivation was a gradual process driven by the accumulation of deleterious missense mutations^24^,^25^. Several amino acid substitutions occurred in the Uox sequence during the primate phylogenetic history. When experimentally introduced in the sequence of a functional Uox, many of these mutations were found to impair protein solubility and/or activity. However, of particular relevance for gene inactivation are the mutations that were fixed in the branch leading to the common ancestor of great and lesser apes (approximately between 30 and 20 Mya). A single missense mutation, Phe→Ser at codon 222, has been precisely identified in the hominoid lineage^25^.

In previous studies, the Urah and Urad enzymes of the urate degradation pathway have been characterized in the model vertebrate zebrafish (*Danio rerio*). With respect to the mammalian counterpart^2^, the isolated proteins from zebrafish demonstrated higher solubility and stability and were amenable to structure-function analysis^26^,^27^. Here we describe the functional characterization of urate oxidase from *Danio rerio (Dr*Uox) through a kinetic assay that allows the determination of the enzymatic activity under physiological pH and salt conditions. The information on the *Dr*Uox structure was used as a framework for the understanding of the catalytic properties of the enzyme in comparison with an artificial variant (F216S) in which the same Phe→Ser substitution that occurred in hominoid evolution was introduced by site-directed mutagenesis.

## Results

### The Phe→Ser mutation and the inactivation of the urate degradation pathway in hominoids

We determined the exon/intron structure of Uox genes and pseudogenes in primates through comparison between the *Macaca fascicularis* protein sequence and primate genomic DNA sequences, allowing for nonsense, frameshift, and splice-site mutations^28^ (Supplementary Fig. S1). To infer the mutations that occurred in the Uox gene in the hominoid lineage we obtained sequence information for the Uox locus from 16 primate species with assembled genomes (6 hominoid and 10 non-hominoid species). The sampling of hominoid species was completed by mapping whole genome sequences (WGS) short reads of four gibbon species (*Hylobates moloch*, *Hylobates pileatus*, *Symphalangus syndactylus*, *Hoolock leuconedys*) on the reference *Nomascus leucogenys* assembled genome. This procedure recovered >90% of the Uox pseudoexonic sequence of the gibbon species (Supplementary Fig. S2), allowing a representation of the eight extant genera of hominoids in the dataset.

**Figure 1:**
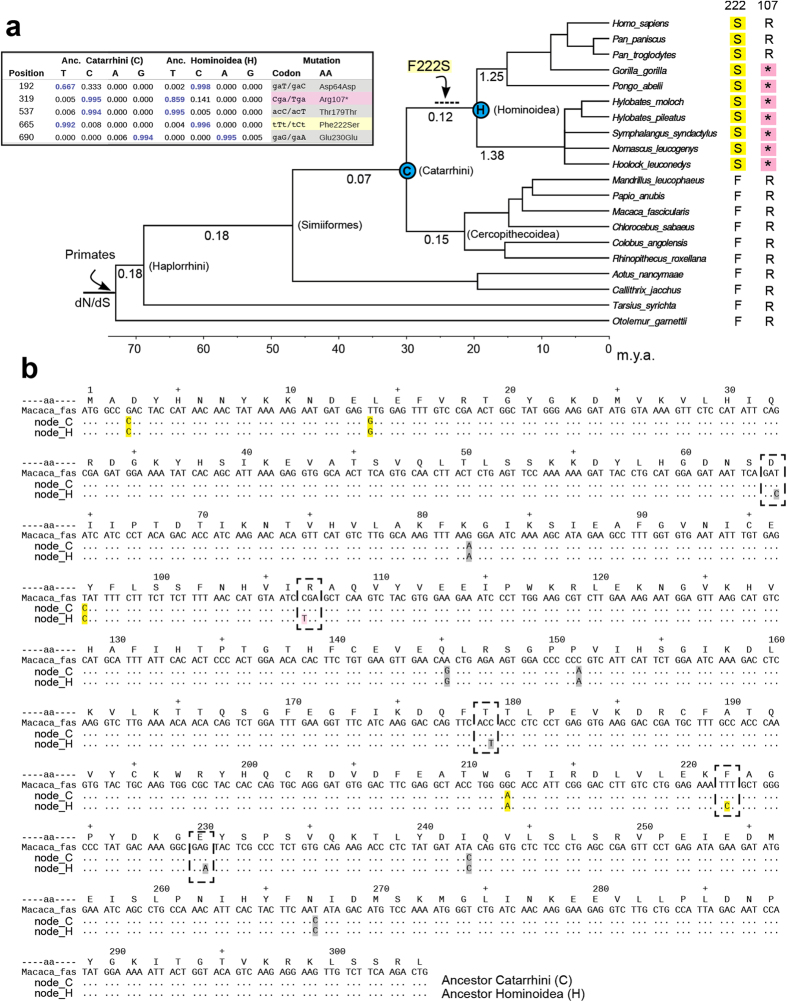
Phe*→*Ser mutation and the inactivation of the urate degradation pathway in hominoids. (**a**) Chronogram of primate phylogeny based on multilocus analysis^58^; a polytomy was introduced to reflect uncertainties in the branching order of gibbon genera^29^,^60^. The tree was used as a framework for Bayesian reconstruction^59^ of ancestral Uox sequences and the ratio of non-synonymous/synonymous substitution rates (dN/dS). Posterior probabilities of nucleotide positions differing between the last common ancestors of Catarrhini and hominoids are shown in the inset. The single missense substitution in the hominoid lineage is indicated on the relevant branch (positions are according with the *M. fascicularis* sequence); the dashed line indicates uncertainty in dating the mutation along the branch. (**b**) The reconstructed Uox sequence of the Cathatrrini last common ancestor (node_C) compared with the sequence of the hominoid ancestor (node_H) using the sequence of *M. fascicularis* as a reference. Synonymous (gray), missense (yellow), and nonsense mutations (pink) with respect to the reference sequence are shown. Mutations which occurred along the branch connecting the two ancestral nodes are framed.

The maximum-likelihood reconstruction of ancestral Uox sequences along the primate tree, identified five accepted mutations of the Uox coding sequence in the branch leading to last common ancestor of extant hominoids (Fig. 1). According to previous analysis^25^, F222S is the only sense mutation which occurred along this branch. However, the analysis of ancestral character states also identified a probable nonsense mutation (R107*) before the split of great and lesser apes (Fig. 1a,b). A stop codon is not found at position 107 of the Uox sequences of human and chimpanzee (Fig. 1a). Nevertheless, the onset of this mutation after the common ancestor of extant hominoids would require three independent mutations in the branches leading to gibbons, *Pongo*, and *Gorilla* (Supplementary Fig. S3). The presence of an Arg codon at this position in human and chimpanzee can be due to reversal of the mutation or to incomplete lineage sorting of a polymorphic allele, a phenomenon commonly observed in ape phylogeny^4^,^29^.

The primate dataset of Uox sequences was also used for an analysis of the selection pressure acting on the Uox gene during evolution, as inferred by calculating the rate of non-synonymous (dN) and synonymous (dS) mutations along the phylogenetic tree. Models with no variation of the dN/dS ratio and models assuming one or more than one persistent variations along branches were compared through the likelihood ratio test. If a single rate variation is assumed along the primate tree, the best likelihood is obtained with a change of dN/dS in the hominoid lineage (Table 1); this 2-rates model is significantly better than the 1-rate model. The model with two independent variations in the gibbon and great apes lineages (3-rates) is slightly better than the 2-rate model, while other models are not significantly better than the 3-rate model. However, these models consistently indicate that the *Uox* gene was under strong selection pressure during primate evolution, including most of the branch leading to hominoids (Fig. 1a). Accordingly, on this branch a large excess of synonymous over sense mutations (3:1) is observed with respect to the proportion of synonymous and non-synonymous sites in the Uox sequence (249:671). Overall, the dN/dS analysis is consistent with relaxation of the selection pressure and pseudogenization of the *Uox* gene near the split of great and lesser ape (about 20 Mya). The F216S variant can thus be considered the last functioning Uox in hominoid evolution.

### Zebrafish urate oxidase (*Dr*Uox) expression and activity assay

**Figure 2:**
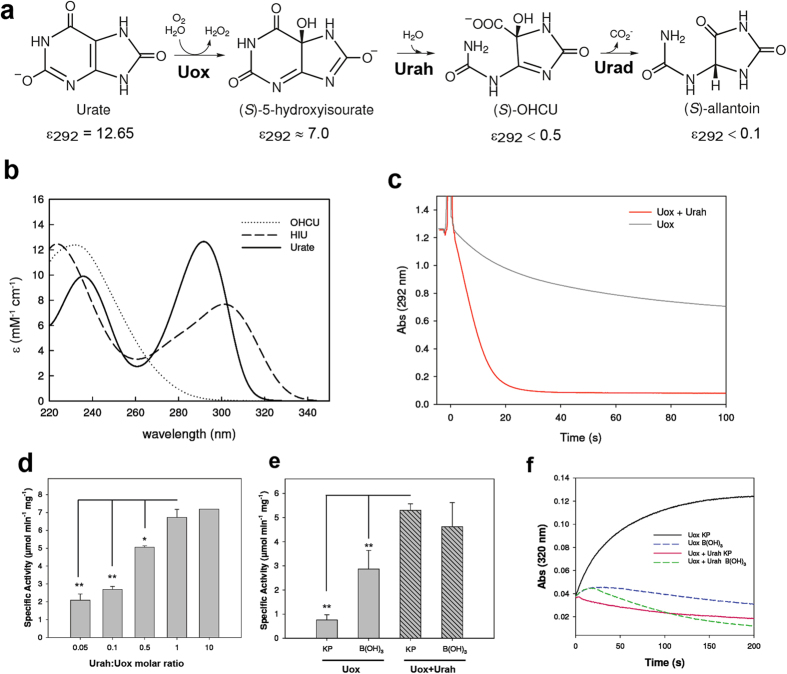
Enzymatic suppression of the spectrophotometric inference in the Uox reaction. (**a**) Scheme of the enzymatic conversion of urate into allantoin; molar extinction coefficients (mM^−1^ cm^−1^) at 292 nm, pH 7.6, are indicated. (**b**) Experimental urate UV spectrum and calculated HIU and OHCU spectra in the region between 220 and 340 nm; approximate spectra were obtained by kinetic analysis^56^ of the time resolved spectra of the Uox reaction (supplementary Fig. S4). (**c**) Decrease of absorbance at 292 nm of 0.1 mM urate with the addition of *Dr*Uox (0.9 μM) in the absence (black curve) and in the presence (red curve) of equimolar *Dr*Urah. (**d**) Uox activity as monitored by decrease of absorbance at 292 nm in the presence of different *Dr*Urah:*Dr*Uox molar ratios. (**e**) Uox activity in potassium phosphate (KP) buffer (100 mM, pH 7.6) and Borate (B(OH)_3_) buffer (50 mM, pH 9.2) in the absence (filled bars) and in the presence (striped bars) of equimolar *Dr*Urah. (**f**) Formation and decay of the HIU product of the Uox reaction monitored by absorbance at 320 nm in KP and B(OH)_3_ buffers in the absence and in the presence of equimolar *Dr*Urah.

The zebrafish (*Danio rerio*) urate oxidase (chr11: 8151055.8158017) encodes a protein of 298 aa with a C-terminal tripetide (ARM) corresponding to the type 1 peroxisome-targeting signal. The unmodified *Dr*Uox coding sequence was overexpressed in *E. coli* and the corresponding protein purified to near apparent homogeneity using a dedicated xanthine-agarose column. The *Dr*Uox activity was measured *in vitro* using a kinetic assay based on the enzymatic suppression of the spectrophotometric interference of the Uox reaction product (Fig. 2).

The direct monitoring of the decay of the urate signal at 292 (or sometimes 293) nm is a widely used assay of the Uox activity^25^,^30^,^31^. However, it was recognized early^14^,^31^ that the unstable product of urate oxidation, 5-hydroxyisourate (HIU), absorbs at this wavelength interfering with the measurement. The extinction coefficient of HIU at 292 nm is about 50% of that of urate (Fig. 2a,b and supplementary Fig. S4), producing a substantial error on the initial velocity estimate (Fig. 2c); moreover, the decay velocity of HIU is influenced by the reaction conditions (i.e. buffer and pH), producing false dependencies of the Uox reaction^14^. To alleviate the interference on the measurement at 292 nm, the Uox assay is often conducted in borate (B(OH)_3_) buffer at high pH, conditions that cause a more rapid decay of HIU^28^,^31^.

The use of HIU hydrolase (Urah) in a physiological potassium phosphate (KP) buffer (100 mM, pH 7.6) enabled the elimination of the interference at 292 nm by catalysing the conversion of HIU to OHCU, a compound that has negligible absorbance at this wavelength (Fig. 2a,b). Urah is a very active enzyme (specific activity: 230 ± 6 μmol min^−1^ mg^−1^), and we found that a Urah:Uox molar ratio of 1:1 (>10-fold excess of enzyme units) is sufficient to eliminate HIU interference and attain the maximum initial velocity (Fig. 2d). In our reaction conditions, this initial velocity was about 7-fold higher than that measured in the absence of Urah in phosphate buffer and 1.5-fold higher than that measured in borate (Fig. 2e), as the use of this nonphysiological buffer did not completely eliminate the HIU interference (Fig. 2f).

At variance with the reaction curve obtained with Uox alone, the curve in the presence of Urah could be fitted with a simple model of enzymatic disappearance of substrate^32^ (Supplementary Fig. S5). The comparison of the decrease in absorbance at 292 in the absence of Urah with the curve resulting from the combination of urate decrease and HIU formation and decay shows that most of the difference between the two reaction progression curves of Fig. 2c can be explained by the HIU interference at 292 nm. However, the experimental kinetics in the absence of Urah is appreciably slower than that predicted based on the absorbance of HIU. In addition, fitting of the kinetics of HIU formation monitored at 320 nm suggested that urate is degraded with the same V_max_ as in the presence of Urah, but with a higher apparent K_M_, consistent with product inhibition. The combination of urate disappearance and HIU formation kinetics with an increased K_M_ reproduced well the experimental behavior of the Uox reaction in the absence of Urah (Supplementary Fig. S5).

### Reaction kinetics and inhibition mechanism of wt *Dr*Uox and F216S mutant

**Figure 3:**
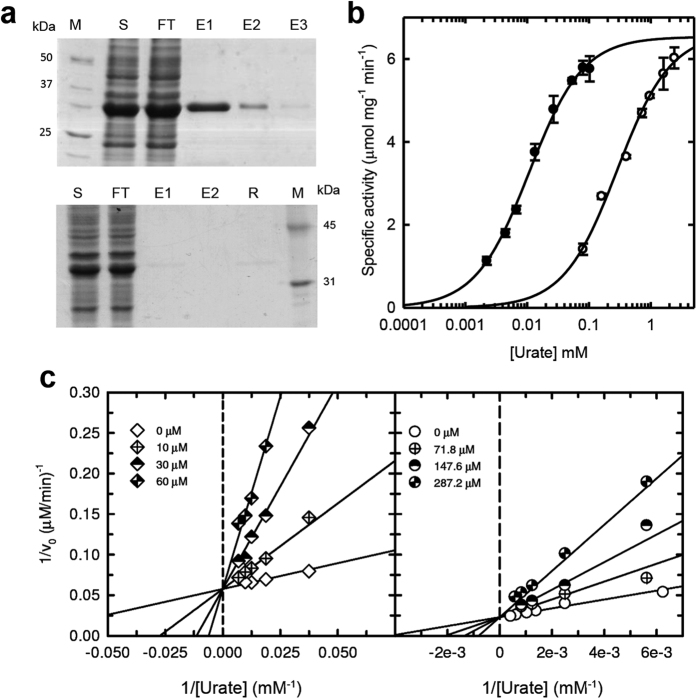
Binding and activity of *Dr*Uox and F21α mutant. (**a**) SDS-PAGE of the protein fractions of xanthine-agarose affinity chromatography for the wt (upper panel) and mutant (lower panel) *Dr*Uox; M: marker; S: soluble cell fraction; FT: Flow-Through; E1-E3: Elution fractions: R: Resin (**b**) Dependence of the initial rate of oxidation on urate concentration of wild type (closed circles) and mutant F216S (open circles) *Dr*Uox; data points were fitted to the Michaelis-Menten equation. (**c**) Dependence of the initial rate of oxidation on urate concentration of wild type (left panel) and mutant F216S (right panel) *Dr*Uox in the presence of increasing xanthine concentrations. Data points were fitted with a global fit to the equation for the competitive inhibition model.

To examine the consequences of the Phe→Ser mutation which occurred in hominoid evolution on the catalytic properties of *Dr*Uox, the F216S mutation (corresponding to F222S in the hominoid sequence) was introduced by site-directed mutagenesis. A difference between the wild-type and mutant proteins emerged during purification; at variance with the wt Uox, the F216S variant was unable to bind the xanthine-agarose column (Fig. 3a). Because of the impossibility of using the specific affinity column for F216S purification, a polyhistidine tag was added to the N terminus of both the wt and mutant proteins to allow purification by metal affinity chromatography. The His-tagged proteins were purified to near apparent homogeneity in a single chromatographic step. Comparison of the untagged and His-tagged wt proteins revealed no substantial differences in the enzymatic activity.

The rate of catalysis of the wt and mutant proteins subjected to the same purification procedure was measured at increasing substrate concentrations using the above-described spectrophotometric assay in the presence of *Dr*Urah. Both enzymes exhibited Michaelis-Menten kinetics (Fig. 3b). The F216S protein had a 25-fold increased K_M_ (280 μM) with respect to the wt protein (11 μM). By contrast, the same turnover number (k_cat_ ≈4 s^−1^) was observed for the two proteins (Table 2). This indicates that the effect of the Phe→Ser mutation on the protein catalytic efficiency depends on a decreased affinity for the urate substrate. Similarly, a decreased affinity for xanthine, a known inhibitor of the Uox reaction^14^, was suggested by the lack of binding of the F216S mutant to the xanthine-agarose column. Xanthine proved to be a competitive inhibitors for the wt and mutant enzymes, affecting the apparent K_M_, but not the maximal velocity of the reaction (Fig. 3c). However, the inhibition constant (K_i_) for xanthine was about one order of magnitude higher for the mutant enzyme with respect to the wild-type (Table 2).

**Figure 4:**
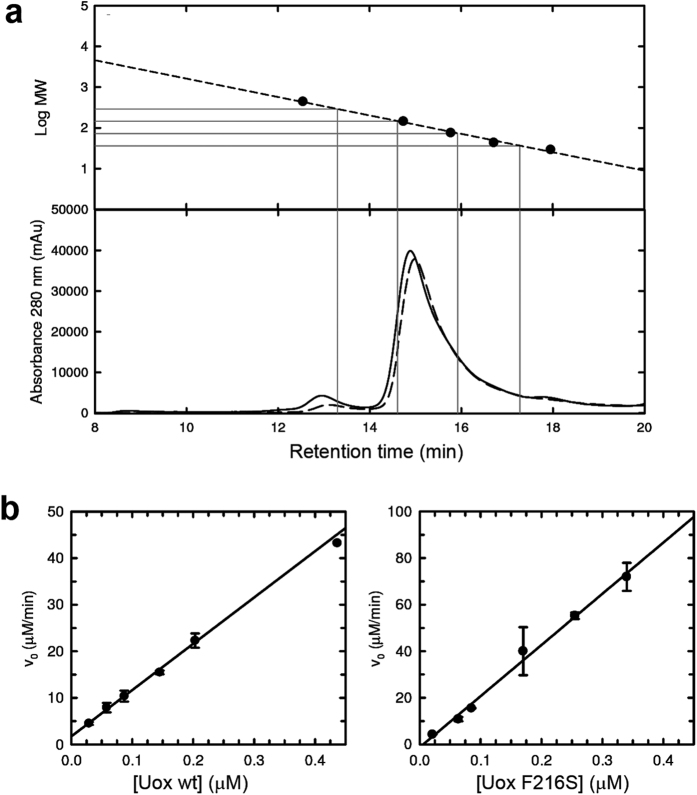
Quaternary states of *Dr*Uox and F216S mutant in solution. **(a**) High-performance size-exclusion chromatography analysis of wt (solid line) and F216S (dashed line) *Dr*Uox; gray dropped lines represent the predicted retention times for the different oligomeric states (from left to right: octamer, tetramer, dimer, monomer). Calibration curve obtained from the separation of standard solutions of ferritin (440 kDa), GAPDH (144.2 kDa), conalbumin (75 kDa), ovalbumin (43 kD) and carbonic anidrase (29 kDa). (**b**) Activities of wild type (left panel) and mutant F216S (right panel) *Dr*Uox tested at increasing concentration of enzyme in the presence of 27 μM and 400 μM urate, respectively; reactions were carried out in the presence of equimolar *Dr*Urah at 25 °C.

No substantial differences in the population of oligomers were observed for the wt and mutant *Dr*Uox, with a large prevalence of the tetrameric assembly for both proteins (Fig. 4a). For both enzymes, we observed a linear dependency of the enzymatic activity on the concentration of the protein in solution (Fig. 4b), thus ruling out a protein concentration-dependent shift in the equilibrium among functionally different quaternary species. However, the F216S mutant appeared to be more prone to thermal inactivation at temperatures ≥40 °C, and less soluble in highly concentrated solutions. When crystallization solutions were prepared, the wt protein could be concentrated up to 10 mg/ml, while the F216S protein started to precipitate at ~2 mg/ml concentration.

### F216 in the *Dr*Uox crystal structure

The crystal structure of *Dr*Uox was obtained at 2.8 Å resolution (Supplementary Table S1) with a continuous electron density from Gly8 to Pro294. Structures of the T-fold urate oxidase have been determined previously for bacterial proteins^30^,^33^,^34^, the fungus *Aspergillus flavus*^15^, and a ‘fossil’ euarchontoglires (primates and rodents) protein reconstructed through phylogenetic inference^25^. The highest structure similarity of the *Dr*Uox monomer is with the fossil euarchontoglires protein (PDB ID 4MB8; rmsd 0.8 Å) and the lowest similarity is with the Uox from *Bacillus* spp. (PDB ID 3WLV; rmsd 2.5 Å).

**Figure 5:**
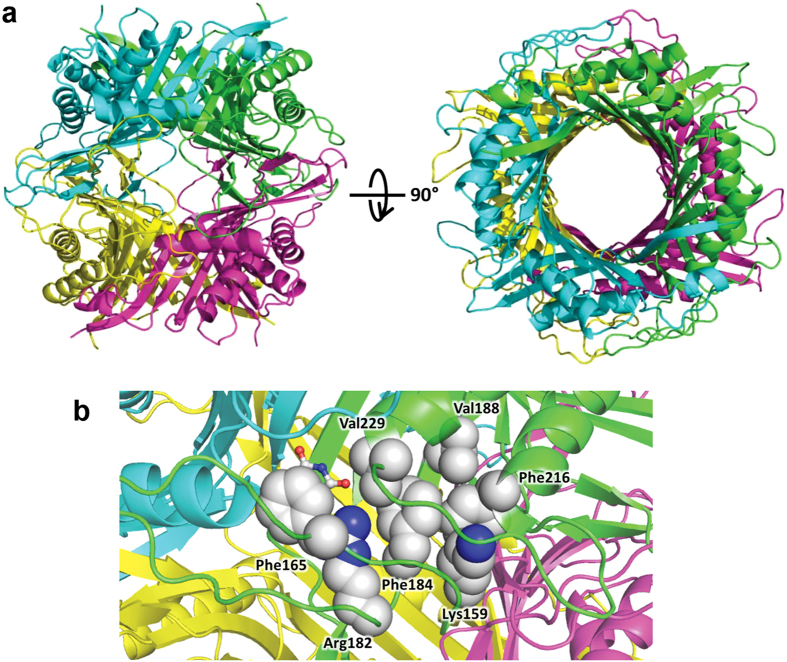
Structure of *Dr*Uox tetramer. (**a**) Side and top view of the *Dr*Uox structure in cartoon representation colored by chains. (**b**) Detail of active site at the interface between chains A (green) and B (cyan); residues involved in a cluster of hydrophobic interactions with F216 are shown as spheres according to their Van der Waals radii. Each residue in the cluster is at <5.5 Å distance from another hydrophobic residue. The urate molecule (sticks) was docked by structural superimposition with the enzyme-substrate complex of *Aspergillus flavus* (PDB id 3BJP).

The *Dr*Uox structure (Fig. 5) shows the typical T-fold with a homotetramer organized as a dimer of dimers joining head-to-head to form the functional tetramer. Each monomer contains two tandem repeats of the T-fold domain forming an antiparallel β_8_-sheet with four main α-helices located at the concave side of the sheet. Two monomers assemble into a dimer by a two-fold axis to form a β_16_α_8_ barrel (Fig. 5a). The main dimerization interface is an antiparallel β-sheet formed by the N-terminal (aa 8–20) and C-terminal (aa 282–293) β strands of two subunits (Supplementary Fig. S6). Several hydrogen bonds involving backbone atoms link the two β strands. Other electrostatic interactions involve the residue side chains of different subunits such as T65-T174 and N68-T175. The identity of such residues is typically conserved in Uox multiple alignments (Supplementary Fig. S7). The tetramerization interface has two main regions of interactions. One region involves mainly side chain interactions (H-bonds and salt bridges) between polar residues such as R28-D273, E37-Y259, K75-S270. This region of interaction comprises a loop between strands β10 and β11 that is conserved in vertebrate proteins, but not in other Uox with known structure. A second region involves a short antiparallel β-sheet formed by part of the strands β5 (aa 125–127) and β7 (aa 157–159). Phenylalanine 216, a residue strictly conserved in Uox alignment, is not part of the tetramer interface, but establishes main chain and side chain interaction with lysine 158 of the tetramer interface (Supplementary Fig. S6).

In the crystal structure, chains A-C and B-D at the tetramer interface are connected by a disulfide bond involving Cys129 (Supplementary Fig. S8), a residue not conserved in Uox sequences (see Supplementary Fig. S7). The geometrical parameters of the disulfide, classify it as having a Left-Handed Staple conformation (+LHStaple), a rare bond characterized by a high strain energy (Supplementary Table 2). SDS-PAGE analysis provided evidence of the presence in solution of a inter-subunit covalent link under non-reducing conditions; however, *Dr*Uox stability, as measured by retention of enzymatic activity at different temperatures, was substantially increased under reducing conditions (Supplementary Fig. S8).

The active sites (four in the tetramer) are located at the dimerization interfaces. F216 is not directly involved in substrate binding, but is involved in a cluster of hydrophobic interactions with active site residues (Fig. 5b). In particular, this cluster comprises conserved residues K159, F184, V188, V229, R182, and F165. Through its guanidino group, R182 establishes a bidentate interaction with the urate substrate. The aromatic ring of F165 forms a stacking interaction with the urate purine ring, while V229 borders the active site cleft and makes a backbone interaction with the substrate. The substitution of F216 with a polar serine residue is expected to perturb this hydrophobic cluster and impact the formation of the Michaelis complex.

## Discussion

Together with HIU hydrolase (Urah) and OHCU decarboxylase (Urad) from the same organism^26^,^27^, the zebrafish urate oxidase characterized here constitutes the enzymatic complement of uricolysis. At variance with the administration of the Uox alone, a pharmaceutical preparation containing such a complete uricolytic system with appropriate chemical modifications (L. Ronda *et al*., manuscript in preparation) would ensure physiological conversion of urate into (*S*)-allantoin and prevent the accumulation of reactive intermediates of urate oxidation.

An unexpected feature of the *Dr*Uox structure was the presence of a disulfide bridge covalently linking different subunits at the tetramer interface. This information is relevant for the *in vitro* activity and the practical use of the enzyme as we conclude that the reduction of the disulfide is likely to occur within the cell. Although this feature has not been previously observed in natural Uox, a disulfide bond has been introduced on purpose at the tetramer interface of a bacterial Uox by site-directed mutagenesis. Interestingly, this engineered protein showed increased thermal stability that was negatively affected by reducing compounds^35^. By contrast, the presence of reducing compounds increased the functional stability of *Dr*Uox. A possible explanation to the different contribution of disulfide bonds to Uox stability is that the bond introduced in *Bacillus* sp. TB-90 is located at the main dimer-dimer interface while in our case it is located in a region that is normally not involved in extensive inter-subunit interactions. Relocation of the disulfide bond in *Dr*Uox by site-directed mutagenesis could be a strategy to improve thermal stability, if required. Evidence in the crystal and in solution indicates that *Dr*Uox is prone to the formation of non-native intra- and inter-chain disulfide bonds. A tendency to the formation of non-native disulfide bonds has been reported for other eukaryotic Uox, and might account in part for the complex mechanism of its irreversible thermal denaturation^36^. The effect of reducing agents suggests that the presence of free thiols is a preferred condition for the *Dr*Uox protein. As for other animal Uox, the zebrafish enzyme has been selected by evolution to function inside peroxisomes, organelles in which the redox state is more reducing than in the cytosol^37^. The reducing conditions of the peroxisome lumen limit the threat of cysteine oxidation and the formation of non-native disulfide bonds, providing a possible rationale for the more frequent usage of this amino acid in eukaryotic Uox (Supplementary Fig. S8).

The addition of the Urah enzyme in the spectrophotometric assay for the Uox reaction allowed elimination of the interference of the HIU product and a more reliable measure of the enzymatic activity in physiological buffers. The modified spectrophotometric assay was used for a detailed comparison of the enzymatic activity of the wt and F216S mutant in order to shed light on the genetic modifications that preceded the loss of the urate degradation pathway in hominoid evolution. We found that this mutation does not change the enzyme turnover number (k_cat_), but increases about 25 times its Michaelis constant (K_M_). Structural and biochemical evidence suggests that the Phe→Ser substitution impacts the formation of the enzyme-substrate complex through the alteration of a cluster of hydrophobic residues involved in substrate binding.

Maximum likelihood reconstruction of ancestral sequences suggests the possible occurrence of a nonsense mutation (R107*) before the split of great and lesser apes (see Fig. 1). A caveat to this conclusion is that the nucleotide substitution model used in the analysis does not account for the hypermutability of the CpG dinucleotide. Several stop codons observed in the Uox pseudogenes of extant hominoids originate from mutation of CGA Arg codons into TGA opal stop codon; vulnerability of methylated cytosine to spontaneous deamination to thymine at CpG sites increases the possibilities of recurrent C→T mutations^4^. This increased mutability is confirmed by the substitutions observed in our hominoid data set: with respect to eleven CpG dinucleotide positions in the reconstructed hominoid ancestor sequence, seven cases of single C→T mutations and two cases of recurrent C→T mutations are observed in the sense strand of extant hominoid sequences. However, the hypothesis of late appearance of a stop codon at position 107, would require independent fixation of the mutation in three lineages. As there are two early nonsense mutations, R18* and R33*, fixed in the Uox sequence of lesser and great apes, respectively^4^, the independent fixation of the R107* mutation should have occurred by random genetic drift. Although this hypothesis cannot be ruled out, the pattern of sense and nonsense codons at position 107 could be explained by a back mutation in the Homo-Pan lineage, or with a single mutation and incomplete assortment of a polymorphic allele. This scenario requires maintenance of the polymorphism for a long time (~10 myr) and thus the presence of balancing selection. Some examples are known of the long-term maintenance of polymorphisms during ape evolution. For instance, the polymorphism at the ABO blood groups is thought to have originated before the ape last common ancestor and been maintained for >20 myr^38^.

Analysis of available genomes indicates that urate oxidase cannot be easily lost in vertebrate organisms. Even uricotelic vertebrates such as birds and some reptiles conserve a Uox gene without obvious inactivating mutations^23^. Among the sequenced mammals, hominoids are the only group with evidence of Uox pseudogenization. The detrimental effect of Uox inactivation in mammals is well demonstrated by the Uox KO mouse, which is affected by perinatal mortality caused by obstructive nephropathy due to urate deposition in the kidney^39^. As a whole, this evidence has suggested that during primate evolution other genes related to purine metabolism changed “to accommodate the increased levels of uric acid”^5^. As demonstrated by the mouse Uox KO phenotype, the renal handling of urate is key to the management of high urate concentrations. Urate homeostasis is regulated by a set of membrane transporters located in the epithelial cells of the kidney (and to a lesser extent of the intestine) involved in secretion and absorption^5^,^6^. In human and apes, 90% of the filtered urate is reabsorbed along the nephron. Lower levels of renal reabsorption have been described in other primates, rodents and the dog, while in other mammals (e.g. pig) urate is not reabsorbed. In organisms that have lost the urate degradation pathway, renal reabsorption maintains high urate concentration in the blood while preserving the kidney function. This is underpinned by the association of nephrolithiasis and kidney failure with loss-of-function mutations of membrane transporters SLC22A12 and SLC2A9 involved in urate reabsorption, as observed in hereditary hypouricemia^40^,^41^.

The Uox gene was under strong and relatively constant purifying selection during most of the primate evolutionary tree, including (at least part of) the branch leading to the last common ancestor of hominoids; the selection pressure relaxed abruptly in the great and lesser apes lineages, with dN/dS values close to the unit, as typical of pseudogenes (see Fig. 1 and Table 1). A similar analysis conducted on the two other genes of the urate degradation pathway, Urah and Urad, shows a comparable trend, with a relaxation of the purifying selection after the split of hominoids. The fact that the inferred dN/dS value for these two genes is still below the unit, could indicate that the pseudogenization of the two other genes of the pathway was consequent to the Uox psuedogenization^23^. In summary, dN/dS analysis does not suggest a progressive decline of the selection pressure acting on genes involved in urate degradation during primate evolution. As already pointed out^25^, this observation is difficult to reconcile with the hypothesis of fixation of deleterious missense mutations in the Uox sequence. The possibility for a deleterious mutation to be fixed by random drift increases in small populations. It should be noted, however, that the ancestral population of the hominoid is estimated to be relatively large, about five time larger than the ancestral population of the genus *Homo*^42^.

Mutations decreasing the Uox catalytic efficiency are expected to increase the *in vivo* concentration of urate. Such mutations could have been perpetuated by evolution in spite of a detrimental effect on the Uox function and the threat posed by the elimination of a poorly soluble molecule, because of the compensatory benefit of high serum urate concentration. Numerous hypotheses on the possible advantages of elevated serum urate levels have been put forward^43^,^44^,^45^, although a clear demonstration of a beneficial role of urate in humans is still lacking. In this scenario, the accumulation of detrimental mutations in Uox, culminating in the complete inactivation of the gene, was the evolutionary force driving a change of purine metabolism in hominoids. Based on biochemical and sequence analysis data, we propose an alternative scenario in which the modification of the renal handling of urate preceded the genetic modifications in the Uox gene. In the presence of increased levels of circulating urate due to renal reabsorption and possibly to changes in the dietary habits^46^, mutations increasing the Uox K_M_ for the substrate would have been neutral or even adaptive. In turn, a renal system able to handle high levels of urate could have permitted the complete dismissal of the degradation pathway by gene inactivation.

It is generally assumed that the kinetic parameters of enzymes, particularly k_cat_ and K_M_, are shaped by evolution according to the organism’s needs^47^,^48^. For some enzymes involved in very important pathways the catalytic efficiency, expressed as k_cat_/K_M_, approximates the highest theoretical values (10^8^–10^9^ M^−1^ s^−1^)^49^. However, the k_cat_/K_M_ values for the large majority of enzymes lie in the range of 10^3^–10^6^ M^−1^ s^−1^. In this respect, Uox is an example of the ‘average’ enzyme —that is an enzyme that is not under strong selective pressure for catalytic efficiency^48^. On the other hand, the magnitude of the Michaelis constant (K_M_) is in correlation with the *in vivo* concentration of the substrates, suggesting evolutionary adaptation of K_M_ values to the reactant concentrations^47^,^50^. This implies that as a consequence of an increased physiological level of a metabolite, mutations increasing the K_M_ (and thus decreasing the enzyme ‘catalytic efficiency’) are not expected to have a deleterious effect on the fitness. The F222S mutation is not observed in extant Uox proteins. However, there are natural Uox enzymes with K_M_ values similar to that observed in the *Dr*Uox mutant. Interestingly, a high K_M_ for urate (~300 μM) has been reported for the enzyme of *Bacillus fastidiosus*, a bacterium specialized for growth in urate-rich media^30^. Also, similarly to the mutant *Dr*Uox, the *B. fastidiosus* protein has a high inhibition constant for xanthine (K_i_ = 41 μM).

Our results suggest that the last functional urate oxidase in hominoid evolution had a rather high Michaelis constant, possibly near to the upper end of the normal range of urate in the human serum. As the goal of an enzymatic therapy should be the maintenance of urate concentrations in a physiological range, a protein with this catalytic property could be considered for a long-term enzymatic treatment with urate oxidase.

## Methods

### Gene cloning and mutagenesis

A cDNA encoding the complete sequence of zebrafish urate oxidase (IMAGE:100059545) was PCR-amplified using a high fidelity thermostable DNA polymerase (Deep Vent DNA polymerase, New England Biolabs) and two sequence-specific primers: an upstream primer (5′-CATATGGCCACTACCTCAAATC-3′) and a downstream primer (5′-GGATCCTTGTCTTCACATTCTG-3′). The amplification product cloned into pNEB193 vector (New England Biolabs) was digested with BamHI and NdeI and subcloned into the expression vector pET11b. The urate oxidase mutant F216S was obtained by site-directed mutagenesis using a high fidelity thermostable DNA polymerase (Pfu Ultra II Fusion HS DNA polymerase, Stratagene) and the primer 5′-CCGTCATTCAAAAGTCTGCAGGACCCTACGATCG-3′ and its reverse complementary. The plasmid pET11b-*Dr*Uox was used as template and the reaction products were treated with DpnI (Stratagene) to digest the parental DNA template. For the production of His-tagged proteins, the sequences encoding *Dr*Uox wild type or F216S mutant were isolated by NdeI-BamHI digestion from the pET11b vector and inserted into pET28b (Novagen), in frame with the sequence coding for the N-terminal 6xHis tag. The resulting expression vector was electroporated into *E. coli* BL21-CodonPlus(DE3) competent cells.

### Protein expression and purification

The expression in *E. coli* BL21 codon plus (DE3) strain in LB medium was induced by adding 1 mM IPTG. After 16 hours incubation at 20 °C, cells were resuspended in 100 mM potassium phosphate (KP) buffer at pH 7.6, in the presence of 300 mM NaCl, 10% glycerol, 0.2 mM PMSF, 0.2 mM benzamidine, 1.5 μM pepstatin A and 1 mg/ml lysozyme and incubated under agitation at 4 °C for 45 minutes. The suspensions were then lysed by sonication and centrifuged at 16000 g for 30 min. For the purification of the untagged *Dr*Uox, soluble cell fraction was applied to a xanthine agarose resin (X3128, Sigma-Aldrich) packed column previously equilibrated with 100 mM KP at pH 7.6. The column was washed with the same buffer and then the protein was eluted with phosphate buffer containing 0.5 mM urate. The His-tagged wt and mutant proteins were purified by affinity chromatography on cobalt-charged resin (Talon, Clontech). A washing step was performed with 50 mM sodium phosphate, 300 mM NaCl, 20 mM imidazole, pH 7.6, prior to eluting the 6xHis-tagged protein from the resin with 50 mM sodium phosphate, 150 mM NaCl, 200 mM imidazole, pH7.6. Purified untagged and His-tagged proteins were diafiltered and concentrated in 100 mM KP, 150 mM NaCl buffer, pH 7.6, by 30 kDa Amicon Ultra-15 centrifugal filter devices (Merck-Millipore). Wt and F216S *Dr*Uox were finally filtered with 0.2 μm filter units to eliminate possible aggregates. Proteins were divided in small aliquots, flash frozen in liquid nitrogen and stored at −80 °C.

### Crystallization, data collection and structure determination

For protein crystallization, a 10 mg/ml solution of untagged *Dr*Uox (10 mM Tris-HCl, pH 7.6, 1 mM EDTA) was diluted 1: 2 with Str. Screen 1 n. 8 (0.1 M Tris-HCl, pH 8.5, 8% PEG 8000). Hanging drops were equilibrated by vapor diffusion against the same reservoir solution. Single crystals were obtained in about a week of incubation at room temperature. Protein crystals were flash frozen in a cryogenic nitrogen stream at 100 K. The dataset was collected at the ID23–2 beam-line of the ESRF Synchrotron Radiation Facility in Grenoble (France). The best crystals diffracted to 2.8 Å resolution. *Dr*Uox crystallizes in the space group P2_1_, with cell parameters ***a*** = *85.341 Å, **b*** = *127.415 Å, **c*** = *132.604 Å, β* = *102.21°*. The asymmetric unit contains two tetramers, corresponding to a Matthews coefficient of 2.68 Å^3^/Da and a solvent content of about 54% of the crystal volume. The correct solution was found using the model of urate oxidase from *Aspergillus flavus* (PDB ID 1R56)^15^. Molecular replacement was performed with Phaser^51^. Refinement was performed using the CNS package^52^ and, in the final steps, Phenix^53^. Several cycles of automatic refinement and manual model building by Coot^54^ reduced the crystallographic R factor to the final value of 0.1976/0.2587 for all the data from 83.3 Å to 2.8 Å resolution. The electron density was quite well defined for all residues from Gly8 to Pro294. The quality of the model was assessed using the Molprobity program (Phenix), and all the criteria were as expected for a structure at this resolution. Data collection and refinement statistics are summarized in Supplementary Table S1.

### Oligomeric state and disulfide bond analysis

The oligomeric state of wt and mutant Uox in native conditions was analyzed on a HPLC-SEC Superdex 200 increase 3.2/300 column (GE Healthcare) by loading about 13 μg of each sample. A calibration curve was determined by running five commercial standards for SEC (blue dextran, ferritin, conalbumin, ovalbumin and carbonic anidrase, GE Healthcare) and the home-made standard glyceraldehyde 3-phosphate dehydrogenase (GAPDH). The elution was monitored at 280 nm. The presence of inter-subunits disulfide bonds was assessed by SDS-PAGE analysis under reducing and non-reducing conditions. Purified *Dr*Uox was alkylated through 30 minutes-incubation with 0.125 M iodoacetamide (IAM, Bio-Rad) at room temperature in the dark and then the excess of IAM was inactivated exposing the solution at light for 30 minutes. Native and alkylated *Dr*Uox were boiled at 95 °C for 5 minutes in denaturing buffer (6% (v/v) SDS, 30% (v/v) glycerol, 0.006% (v/v) Bromophenol Blue, 0.185 M Tris HCl, pH 6.8) in reducing (15% β-mercaptoethanol) and non reducing conditions. All the samples were loaded on 12% SDS-PAGE. The analysis of the geometrical parameters of the disulfide bond was performed with the UNSW online tool^55^ based on the atomic coordinates of *Dr*Uox. The number of cysteines in Uox proteins of different taxonomic divisions was determined for 1385 sequences clustered at 80% similarity with the cdhit program using the pepstat program of the Emboss package. Data were plotted using the geom_boxplot and geom_jitter functions of the ggplot2 library of the R package.

### Activity assays

Catalytic activity measurements were carried out with a Cary 400 Varian UV-visible spectrophotometer with a thermostated cell-holder, at 25 °C. Reactions were started with the addition of the enzyme to a solution of 100 mM KP, pH 7.6, in the presence of different concentrations of urate as substrate. Reaction rates were determined in the presence of 1:1 molar ratio of Urah from zebrafish^26^ to avoid the interference of the Uox reaction product, 5-hydroxyisourate. The activity was monitored at the fixed wavelength of 292 nm, corresponding to the absorbance peak of urate, except for the dependence of F216S activity on xanthine concentration, registered at 302 nm in order to reduce the absorption interference of xanthine. Enzymatic activity dependencies on increasing substrate concentrations in the presence of different xanthine concentrations were globally fitted to the equation for competitive inhibition.

### Calculation of HIU and OHCU spectra and time course kinetic analysis

Approximate absorption spectra of the unstable intermediate HIU and OHCU were obtained by kinetic analysis of the time-dependent spectral evolution of 0.1 mM urate in the presence of Uox and Urad, or Uox and Urah, respectively. The time- dependent spectra were fitted to a sequential model of intermediate formation and decay using the fitModel function implemented in TIMP library^56^ of the R package. Time course kinetic analysis of the Uox reaction in the presence and in the absence of Urah was conducted by fitting single wavelength kinetics to the Schell-Mendoza equation^32^ using the Lambert-W function implemented in the Gnu Scientific Library (gsl) of the R package.

### Reconstruction of primate Uox sequences from assembled genomes and short sequence reads

Genomic regions containing the Uox sequences were identified by homology searches (tblastn) in assembled genomes of the primate division using the reference protein sequence of *Macaca fascicularis* as a query. Portions encompassing 10 k nt. upstream and downstream the coding or pseudo-coding sequence boundaries were extracted for further analysis. Short SOLID reads (50 nt.) of four gibbon species (*Hylobates moloch*, *Hylobates pileatus*, *Symphalangus syndactylus*, *Hoolock leuconedys*) with no assembled genomes^29^ were downloaded from the ncbi Sequence Read Archive (http://www.ncbi.nlm.nih.gov/sra) and converted to basespace using the fastq-dump utility of the SRA toolkit. The reads were then mapped on the reference *N. leucogenys* genome using the BBmap program (http://bbmap.sourceforge.net/). To facilitate the identification of the reads mapping to the Uox gene, the corresponding region of *N. leucogenys* (CDS ± 10 k nt.) was included as separated sequences in the reference assembly and masked in the chromosome sequences. Reads mapped to the Uox locus were filtered from the SAM format output of bbmap using Linux grep and converted to sorted BAM format using SAMtools (Ver. 1.2) (http://samtools.sourceforge.net/). Sequence variation with respect to the reference sequence was determined using Bcftools (Ver 1.2), and a consensus sequence of mapped reads was obtained with the vcfutils.pl utility. The Genewise program^28^ was used to reconstruct the exonic and intronic structures of genes or pseudogenes. Genewise was run with search parameters adapted for pseudogene reconstruction: the probability of indels and substitutions was increased (-indel 1e-3 -susbs 1e-3) and a splice site model was used (-nosplice_gtag) instead of the default flat model to allow for splice site mutations. Also, the gap extension penalty was increased (-e 5) to avoid the insertion of gaps at the intron/exon boundaries in the presence of sub-optimal splice sites.

### Reconstruction of ancestral sequences and dN/dS analysis

The Uox coding and pseudo-coding sequences were aligned with MACSE using different alignment parameter for gene and pseudogenes^57^. Ancestral sequences were reconstructed based on a rooted primate ultrametric tree^58^ and the alignment of extant Uox sequences using the Bayesian method (RateAncestor = 1) implemented in the baseml program of the PAML package^59^. Ancestral probabilities were calculated using a local molecular clock model assuming a long-term rate variation in correspondence with the hominoid lineage. The rates of non-synonymous (dN) and synonymous substitutions (dS) were calculated along the branches of the primate tree using the PAML codeml program. Models assuming persistent variations of dN/dS ratios on different branches of the primate tree were compared by the Likelihood-ratio (LTR) test.

## Additional Information

**Accession codes:** The atomic coordinates of zebrafish urate oxidase have been deposited at the Protein Data Bank with ID code 5M98.

**How to cite this article**: Marchetti, M. *et al*. Catalysis and Structure of Zebrafish Urate Oxidase Provide Insights into the Origin of Hyperuricemia in Hominoids. *Sci. Rep.* **6**, 38302; doi: 10.1038/srep38302 (2016).

**Publisher's note:** Springer Nature remains neutral with regard to jurisdictional claims in published maps and institutional affiliations.

## Supplementary Material

Supplementary Information

## Figures and Tables

**Table 1:** Parameter estimates under models of variable dN/dS ratios of the *Uox* gene among primate lineages.

Model	dN/dS on branches^a^								
	Haplorrhini	Simiiformes	Catarrhini	Cercopithecini	Hominoidea	Hylobatidae	Hominidae	Log likelihood	LRT
H0: (1 rate)	0.33	=Haplor.	=Haplor.	=Haplor.	=Haplor.	=Haplor.	=Haplor.	−2881.94	
H1: (2 rates)	0.17	=Haplor.	=Haplor.	=Haplor.	1.17	=Homino.	=Homino.	−2856.90	1.5E-12^b^
H2: (3 rates)	0.17	=Haplor.	=Haplor.	=Haplor.	=Haplor.	1.25	1.38	−2854.75	0.04^c^
H3: (4 rates)	0.17	=Haplor.	=Haplor.	=Haplor.	0.12	1.26	1.38	−2854.69	0.72^d^
H4: (7 rates)	0.18	0.18	0.07	0.15	0.12	1.26	1.38	−2854.25	0.83^e^

^a^dN/dS on branches leading to the common ancestor of the indicated lineages in accordance with the tree shown in Fig. 1.

^b^H1 vs. H0; df = 1.

^c^H2 vs. H1; df = 1.

^d^H3 vs. H2; df = 1.

^e^H4 vs. H3; df = 3.

**Table 2:** Kinetic and inhibition constants for wt and mutant *Dr*Uox.

	Urate			Xanthine
	K_M_ (μM)	k_cat_ (s^−1^)	k_cat_/K_m_ (M^−1^ s^−1^)	K_i_ (μM)
*Wild Type*	11 ± 1	3.95 ± 0.09	3.6 * 10^5^	4.3 ± 0.27
*F216S*	284 ± 31	3.99 ± 0.1	1.4 * 10^4^	59.2 ± 8.2
