# Supplementary material for: Catalysis and Structure of Zebrafish Urate Oxidase Provide Insights into the Origin of Hyperuricemia in Hominoids
Source: Sci Rep. 2016 Dec 6;6:38302. doi: 10.1038/srep38302 (PMC5138847; doi:10.1038/srep38302)
Supplement: Supplementary Information [file srep38302-s1.pdf]

Catalysis and Structure of Zebrafish Urate Oxidase Provide Insights into the Origin of Hyperuricemia in Hominoids

Marialaura Marchetti[1], Anastasia Liuzzi[1], Beatrice Fermi[1], Romina Corsini[1], Claudia Folli[2], Valentina Speranzini[1], Francesco Gandolfi[1], Stefano Bettati[3], Luca Ronda[3], Laura Cendron[4], Rodolfo Berni[1], Giuseppe Zanotti[4], and Riccardo Percudani[1].

[1]Department of Life Sciences, University of Parma, 43124 Parma, Italy; [2]Department of Food Science University of Parma, 43124 Parma, Italy; [3]Department of Neurosciences, University of Parma, 43124 Parma, Italy, and the [4]Department of Biology, University of Padova, 35121 Padova, Italy.

SUPPLEMENTAL TABLES

Table S1. **Statistics on *Dr*Uox data collection and refinement.**

Table S2. **Geometry of the left-handed disulfide bridge in *Dr*Uox.**

SUPPLEMENTAL FIGURES

Figure S1. **Reconstruction of the exonic and intronic structure of Uox psuedogenes**.

Figure S2. **Gibbon Uox pseudo-coding sequences obtained by mapping short reads into the *Nomascus leucogenys* reference genome**.

Figure S3. **Hypothetical scenarios for the evolutionary origin of the Uox R107* mutation**.

Figure S4. **Kinetic analysis of the time-dependent spectra of the Uox reaction**.

Figure S5. **Fitting of the Uox reaction progression curves**.

Figure S6. **Dimer and inter-dimer interfaces in *Dr*Uox**.

Figure S7. **Multiple alignment of Uox with known structure.**

Figure S8**. *Dr*Uox disulfide bonds in the crystal and in solution**.

Table S1. **Statistics on *Dr*Uox data collection and refinement**.

**X-ray data**	
Space group	$P2_1$
Cell parameters [Å, °]	***a***= *85.34*, ***b***=*127.41*, ***c***=*132.60*, *β=102.21*
Resolution (Å)	83.3-2.8 (2.95-2.80)
Independent reflections	64855 (8859)
Multiplicity	3.5 (3.4)
Completeness (%)	94.9 (89.0)
$\langle I/\sigma(I) \rangle$	5.2 (2.2)
R_{merge}	0.168 (0.427)
**Refinement**	
Protein atoms /solvent	18584 / 101
Mean B value (Å^2)	30.56
R_{cryst} / R_{free}	0.204 / 0.242
**Geometry**	
Ramachandran favored	98.1%
Ramachandran allowed	1.9%
Ramachandran outliers	0%
Rotamer outliers	1.9%
Rmsd on bond length [Å], angles (°)	0.014, 1.69

A wavelength of 0.87260 Å was used; 180 rotations of 1° each were performed. Values in parentheses are for reflections in the highest-resolution shell

Table 2. **Geometry of the left-handed disulfide bridge in *Dr*Uox**.

	**DSE**[a] **(kj mol^{-1})**	**Distance (Å)**			**Angle (deg)**		**Torsion angle (deg)**		
**geometry parameter**		C_α --- C_α	C_β --- C_β	S_γ --- S_γ	C_α --- C_β --- S_γ	C_β --- S_γ --- S_γ	X_1	X_2	X_3
***Dr*Uox**	45	5.5	4.8	2.05	114.7	114.8	169 (trans+)	94.2 (gauche+)	-141.6 (trans-)
**Reported average values**[b]	18.1 (11.4-24.8)	5.8 (5.5-6.1)							

[a]Dihedral strain energy (DSE).

[b]Mean and 95% confidence interval for 29 LHStaple disulfide bridges [55].

A

```
Uox_Mf             1 MADYHNNYKK                           NDELEFVRTGYGKDMVKVLHIQRDGKYHSIKEVATSVQLTLSSKKDYLHGDNSDIIPTDTIKNTVHV
                     MA YHNNYKK                           NDE+EFVRTGYGK+MVKVLHIQ DGKYHSIKEVATSVQLTLSSKKDYLHGDNSDIIPTDTIKNTVHV
                     MAHYHNNYKK                           NDEVEFVRTGYGKEMVKVLHIQXDGKYHSIKEVATSVQLTLSSKKDYLHGDNSDIIPTDTIKNTVHV
gi|568815597     101 agctcaataaGTGAGAC  Intron 1       CAGaggggtgcagtgagagagccactggatcaaagggatgccacataagtccggatgaacagaaaaagcg
                     tcaaaaaaaa<0-----[131    :  13120]-0>aaatattgcgagaattattatagagaaagtaatccctatctgcaaaatagaacattccactaactat
                     gccctcctag                           ttgggtcatctggagaatcttgataatcctaggatagattatcaatcgtattaccctacccgcattc

Uox_Mf            78 LAKFKG                           IKSIEAFGVNICEYFLSSFNHVIRAQVYVEEIPWKRLEK                           NGVKH
                     LAKFK                            IKSIEAFGVNICE+FLSSFNHVIRAQVY+EEIPWK+L K                           NGVKH
                     LAKFKE                           IKSIEAFGVNICEHFLSSFNHVIRAQVYMEEIPWKHLGK                           NGVKH
gi|568815597   13322 tgatagGTATGTG  Intron 2       CAAaaaaggtggaatgctctttacgacgcgtaggactaccgaGTTAACT  Intron 3       CAGaggac
                     tcataa<0-----[13340  :  15823]-0>tagtactgtatgaattcctaattgcatataatcgaatga<0-----[15941  :  16039]-0>agtaa
                     gagtaa                           cacaacttgtttgtttttctacataccgaactggttag                            tatgt

Uox_Mf           128 VHAFIHTPTGTHFCEVEQLRS                              PPVIHSGIKDLKVLKTTQSGFEGFIKDQFTTLPEVKDRCFATQVYCKWRYHQC
                     VHAFIHTPTGTHFCEVEQLRS                              P VIHSGIKDLKVLKTTQSGFEGFIKDQFTTLPEVKD CFATQVYCKWRYHQC
                     VHAFIHTPTGTHFCEVEQLRS          G:G[gga]            PQVIHSGIKDLKVLKTTQSGFEGFIKDQFTTLPEVKDXCFATQVYCKWRYHQC
gi|568815597   16055 gcgtacacagacttgggccaaGGTAAGAA  Intron 4       CAGGAccgactgaagcagtaaactgtggtaagctaaccggagtttgacgttatctcct
                     tacttacccgcatgataatgg <1-----[16119  :  25703]-1>  cattacgtaatattaccacgtagttaaatcctcataaggtccatagaggaaag
                     ctattctctaacctataggat                              cactttacaccgcgaaagtatatccgcgctcctgggcactccagccggcccgc

Uox_Mf           203 RDVDFEAT                              GTIRDLVLEKFAGPYDKGEYSPSVQKTLYDIQVLSLSRVPE
                     RDVDF+AT                               TIRDLV+EK AGPYDKGEY  SVQKTL DIQVLSLSRVP
                     RDVDFKAT           W:W[tgg]           DTIRDLVMEKSAGPYDKGEYLTSVQKTLCDIQVLSLSRVPA
gi|568815597   25865 agggtagaTGGTATGAA  Intron 5       TAGGgaacgcgagatggctgaggttatgcaactgacgctcacgcgGTAAGTT  Intron 6
                     gatatacc  <2-----[25891  :  27904]-2> actgatttaaccgcaaagaatcctaactgatattctggtcc<0-----[28029  :  30191]
                     gtgccgtc                              cctgctcggattgctcatacgctgggccttcggccgcattg

Uox_Mf           253    IEDMEISLPNIHYFNIDMSKMGLINKEE                           VLLPLDNPYGKITGTVKRKLSSRL
                        IEDMEISLPNIHYFNIDMSKMGLINKEE                           VLLPLDNPYGKITGTVKRKLSSRL
                        IEDMEISLPNIHYFNIDMSKMGLINKEE                           VLLPLDNPYGKITGTVKRKLSSRL
gi|568815597   30189 CAGaggagaaccaacttaagataagcaaaggGTAAGAG  Intron 7       CAGgtcctgactgaaagagaaatttac
                     -0>taatatgtcataatatatcatgttaaaa<0-----[30276  :  32436]-0>tttctaacagatcgctagatccgt
                        aatgaccgactccccacgcagtgccgag                           cggaactataatttacggggtaag
```

B

```
Uox_Mf             1 MADYHNNYKK                           NDELEFVRTGYGKDMVKVLHIQRDGKYHSIKEVATSVQLTLSSKKDYLHGDNSDIIPTDTIKNTVHV
                     MA YHNNYKK                           NDE++FV TGYGKDMVK LHIQRDGKYHSIKEV TSVQLTLSSKKD LHGDNSDIIPTDTIKNTVHV
                     MAHYHNNYKK                           NDEVKFVXTGYGKDMVKDLHIQRDGKYHSIKEVVTSVQLTLSSKKDDLHGDNSDIIPTDTIKNTVHV
gi|429476287     101 agctcaataaGTGAGAC  Intron 1       CAGagggatgtagtgagagagccaccggatcaaagggatgccacataaggccggatgaacagaaaaagcg
                     tcaaaaaaaa<0-----[131    :  13113]-0>aaatattgcgagaattaatatagagaaagtaattcctatctgcaaaatagaacattccactaactat
                     gccctcttag                           ttgggtcatctggtgaatcttgataatcctaggatagattgtcaatcgtattaccctacccgcattc

Uox_Mf            78 LAKFKG                           IKSIEAFGVNICEYFLSSFNHVIRAQVYVEEIPWKRLEK                           NGVKH
                     LAKFKG                           IKSIEAFGVNICE+FLSSFNHVI AQVY+EE PWK LEK                           NGVKH
                     LAKFKG                           IKSIEAFGVNICEHFLSSFNHVIXAQVYMEE!PWKCLEK                           NGVKH
gi|429476287   13315 tgatagGTATGTG  Intron 2       CAGaaaaggtggaatgctctttacgatgcgtagg2ctatcgaCTTAACT  Intron 3       CAGaggac
                     tcatag<0-----[13333  :  15841]-0>tagtactgtatgaattcctaattgcatataa cgagtaa<0-----[15958  :  16056]-0>agtaa
                     gggtaa                           cacaacttgtttgtttttctacataccgaa tggttag                            tatgt

Uox_Mf           128 VHAFIHTPTGTHFCEVEQLRS                              PPVIHSGIKDLKVLKTTQSGFEGFIKDQFTTLPEVKDRCFATQVYCKWRYHQC
                     VHAFIHTPTGTHFCEVEQLRS                               PV HSGIKDLKVLKTTQSGF+GFIKDQF TLPEVKDRCFATQVYCKWRYHQC
                     VHAFIHTPTGTHFCEVEQLRS          G:G[gga]            HPV!HSGIKDLKVLKTTQSGFKGFIKDQFITLPEVKDRCFATQVYCKWRYHQC
gi|429476287   16072 gcgtacacagacttgggccaaGGTAAGAA  Intron 4       CAGGAccg4ctgaagcagtaaactgtagtaagctaaccggagcttgacgttatctcct
                     tacttacccgcatgataatgg <1-----[16136  :  26532]-1>  act acgtaatattaccacgtagttaaattctcataaggtccatagaggaaag
                     ctattctctaacctataggat                              cac ttacaccgcgaaagtatatccgcgctcctgggcactccagccggcccgc

Uox_Mf           203 RDVDFEAT                              GTIRDLVLEKFAGPYDKGEYSPSVQKTLYDIQVLSLSRVPE
                     RDVDFEAT                               TIRDLVLEK AGP DKGEYSPSVQKTLYDIQVLSLSRVPE
                     RDVDFEAT           W:X[tga]           DTIRDLVLEKSAGPSDKGEYSPSVQKTLYDIQVLSLSRVPE
gi|429476287   26695 agggtggaTGGTATGAA  Intron 5       TAGAgaacgcgcgatggctgaggttctgcaactgacgctcacgcgGTAAGTT  Intron 6
                     gatatacc  <2-----[26721  :  28716]-2> actgatttaaccgccaagaaccctaactaatattctggtca<0-----[28841  :  30996]
                     gtgccgtc                              cctgctcggattgctcacacgctgggccttcggccgcattg

Uox_Mf           253    IEDMEISLPNIHYFNIDMSKMGLINKEE                           VLLPLDNPYGKITGTVKRKLSSRL
                        IED + SLPNIHYFNIDMSKMGLINKEE                           VLLPLDNPYGKITGTVKRKLSSRL
                        IEDTKSSLPNIHYFNIDMSKMGLINKEE                           VLLPLDNPYGKITGTVKRKLSSRL
gi|429476287   30994 CAGaggaaaaccaacttaagataagcaaaggGTAAGAG  Intron 7       CAGgtcctgactgaaagagaaatttac
                     -0>taacaggtcataatatatcatgttaaaa<0-----[31081  :  33202]-0>tttctaacagatcgctagatccgt
                        aatgaccgactccccacgtagtgccgag                           cggaactataatttacggggtaag
```

Figure S1. **Reconstruction of the exonic and intronic structure of Uox psuedogenes**. Examples of the genewise analysis of the Uox locus in (**A**) *Homo sapiens* and (**B**) *Nomascus leucogenys*. The comparison was performed between the Uox protein sequence of *Macaca fascicularis* (Uox_Mf) and genomic DNA sequences from *H. sapiens* Chr1 (gi|568815597 c84397931-84354556) and *N. leucogenys* Chr12 (gi|429476330 92198215-92166054). The search parameters were adapted to identification of pseudogenes (-nosplice_gtag, -indel 0.001, -subs 0.001, -e 5). Inferred nonsense, frameshift, and splice site mutations are indicated by pink, blue, and gray arrows, respectively.

```
                   *        20         *        40         *        60         *        80         *       100         *       120
N_leucogen : atggcccactaccataacaattataaaaagaatgatgaggtgaagtttgtctgaactggctatgggaaggatatggtaaaagatctccatattcagcgagatggaaaatatcacagcatt : 120
H_moloch   : ....................c.......................a........................................................................... : 120
H_pileatus : ................................................................nnnnnnnnnnnnnnnnnnnnnnnn................................ : 120
Hool_leuco : ....................c.............................................................t..................c.................. : 120
S_syndacty : .........c..........c...........c........................................c........t..................................... : 120

                   *       140         *       160         *       180         *       200         *       220         *       240
N_leucogen : aaagaggtggtaacttcagtgcaacttactctgagttccaaaaaagatgacctgcatggagataattcagacatcatccctacagacaccatcaagaacacagttcatgtcttggcgaag : 240
H_moloch   : ..........c.....................................t.......................g..........................................n.... : 240
H_pileatus : ..........c.....................................t.......................g..................nnnnnnnnnnnnn................ : 240
Hool_leuco : ..........c.....................................t............................................................c.......... : 240
S_syndacty : ..........c.............................c.......t.......................g............................................... : 240

                   *       260         *       280         *       300         *       320         *       340         *       360
N_leucogen : tttaaaggaatcaaaagcatagaagccttttggtgtgaatatttgtgagcatttctttcttcttttaaccatgtaatctgagctcaagtctacatggaagaaatccttggaagtgtcttg : 360
H_moloch   : .....................................................................................n.......g...................c...... : 360
H_pileatus : .....................................................................................nn......g...................c...... : 360
Hool_leuco : .............................................................................................g...................c...... : 360
S_syndacty : ...........................................................................................nnnnnnnnnnnnnnnnnnnngtn...... : 360

                   *       380         *       400         *       420         *       440         *       460         *       480
N_leucogen : aaaagaatggagttaagcatgtccatgcatttattcacactcccactggaacacacttctgtgaagttgaacagctgagaagtggacacccagtcatttcattctggaatcaaagacctc : 480
H_moloch   : .....nnnnnnnnnnnnnnnnnnnnnnnnnnnnnnnnnnnnnnnnnnnnn.....................................c................................ : 480
H_pileatus : ..................................nnnnn................................................c................................ : 480
Hool_leuco : .......................................................................................c................................ : 480
S_syndacty : ....................................................................................ac.c..-............................. : 479

                   *       500         *       520         *       540         *       560         *       580         *       600
N_leucogen : aaggtcttgaaaacaacacagtctggatttaaaggtttcatcaaggaccagttcattaccctccctgaggtgaaggaccgatgctttgccacccaagtgtactgcaagtggcgctaccac : 600
H_moloch   : ............................................................................................nnnnnnnnnnnnnnn............. : 600
H_pileatus : .....................................................................................................nnnnnnnnnnnnnnnnnn. : 600
Hool_leuco : ...c......................................................t..................................t.......................... : 600
S_syndacty : ..a............................................................................................................t........ : 599

                   *       620         *       640         *       660         *       680         *       700         *       720
N_leucogen : cagtgcagggatgtggacttcgaggctacctgagacaccattcgggaccttgtcctggagaaatctgctgggccctctgacaaaggcgaatactcgccctctgtgcagaagaccctctat : 720
H_moloch   : ........................................................................................................................ : 720
H_pileatus : ......nnnnnnnnnnnnnnnnnnnnnnnnnn............................................g..........................................n : 720
Hool_leuco : ............................................................................a........................................... : 720
S_syndacty : ..................................g.........................................a........................................... : 719

                   *       740         *       760         *       780         *       800         *       820         *       840
N_leucogen : gatatccaggtgctctccctgagccgagttcctgagatagaagatacgaaaagcagcctgccaaacattcactacttcaacatagacatgtctaaaatgggtctgatcaacaaggaagag : 840
H_moloch   : .................................n..........................................................NNNNNNNNNNNNNNNNNNNNNNNNNNNN : 840
H_pileatus : nnnnnnnnnnnnnn....................................................................g..................................... : 840
Hool_leuco : ..c.........................c.................t...................................c..................................... : 840
S_syndacty : ..............................................t.g...t.............................c..................................... : 839

                   *       860         *       880         *       900         *
N_leucogen : gtcttgctgccattagacaatccatatggaaaaattactggtacagtcaagaggaagttgtcttcaagactg : 912
H_moloch   : ........................................................................ : 912
H_pileatus : ....................................................................nnnn : 912
Hool_leuco : .......................................................................n : 912
S_syndacty : ........................................................................ : 911
```

Figure S2. **Gibbon Uox pseudo-coding sequences obtained by mapping short reads into the *Nomascus leucogenys* reference genome**. In the sequence alignment, dot and nucleotide symbols indicate, respectively, positions identical or variant with respect to the *N. leucogenys* reference sequence; ‘n’ symbols represent positions not covered by the mapping procedure.

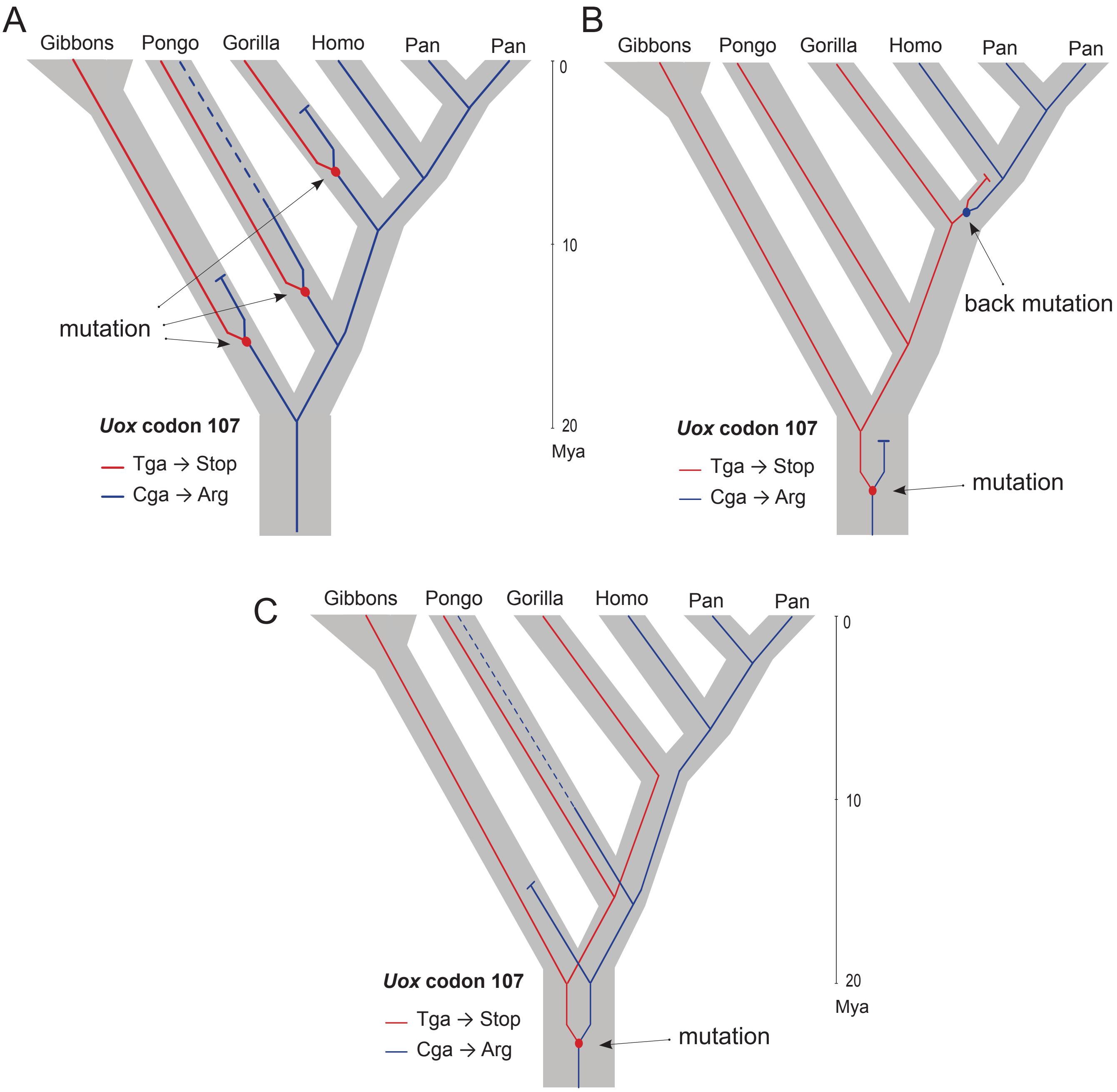

Figure S3. **Hypothetical scenarios for the evolutionary origin of the Uox R107* mutation**. (**A**) three independent mutations in the gibbon, *Pongo*, and *Gorilla* lineages. (**B**) Mutation in the hominoid lineage followed by a back mutation in the *Homo-Pan* lineage. (**C**) Incomplete lineage sorting of a polymorphic allele originated in the hominoid lineage; alternative alleles can be either lost (e.g. gibbons) or reduce their frequency (e.g. *Pongo*) after speciation, or be not inherited during speciation (e.g. *Gorilla*, *Homo-Pan*). Note that this hypothesis requires that the site remains polymorphic for at least 10 Myr.

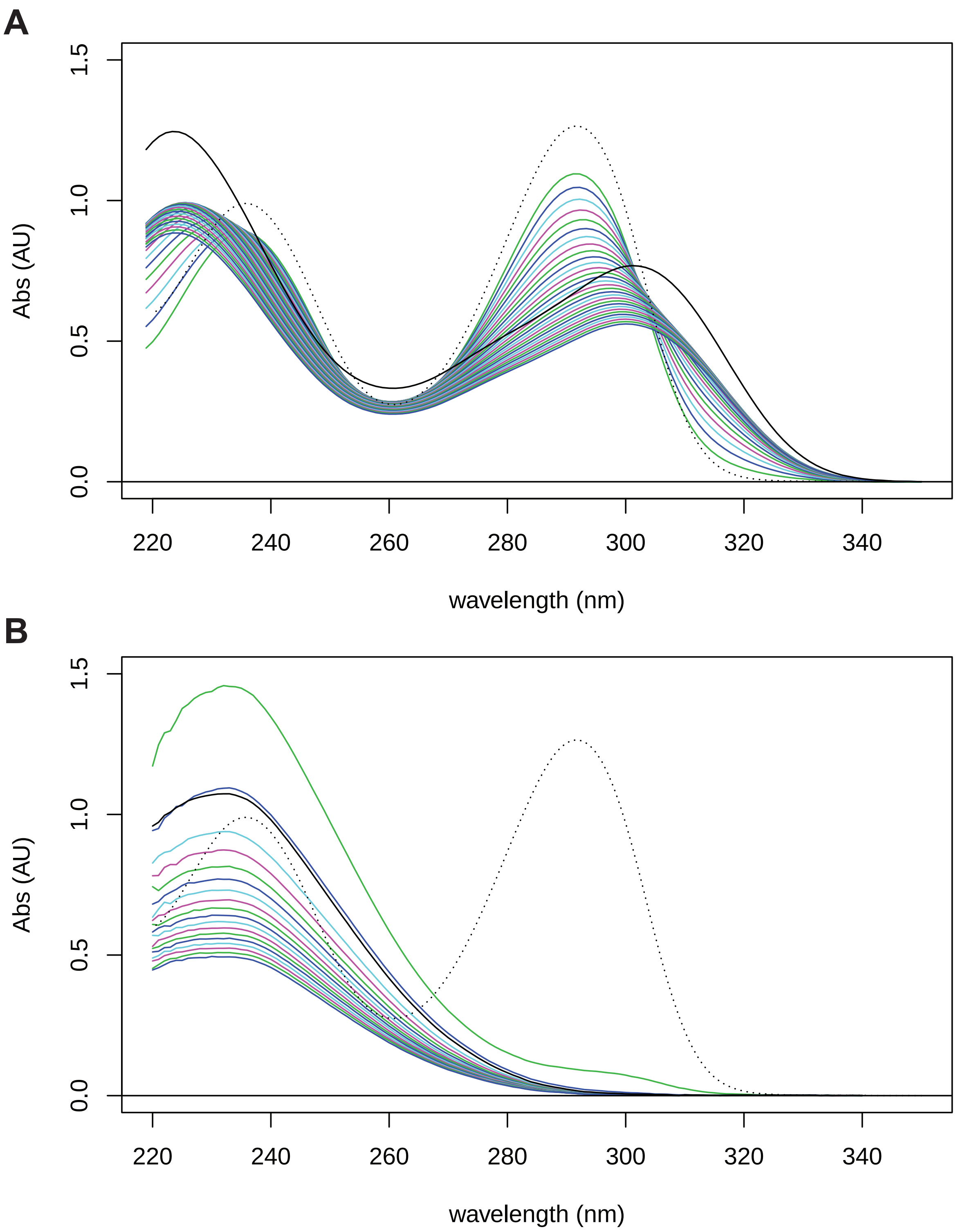

Figure S4. **Kinetic analysis of the time-dependent spectra of the Uox reaction**. Colored continuous lines represent the time evolution of the absorbance spectra of solutions containing urate (0.1 mM) plus the Uox and Urad (panel A), or the Uox and Urah enzymes (panel B). The dotted line is the experimental spectrum of urate. Approximate spectra (black lines) for HIU (panel A) and OHCU (panel B) were obtained by kinetic analysis of the time-dependent spectral series using a two-component sequential kinetic model (HIU formation and decay) in panel A and a single component kinetic model (OHCU decay) in panel B. The analysis was conducted with the TIMP library of the R package.

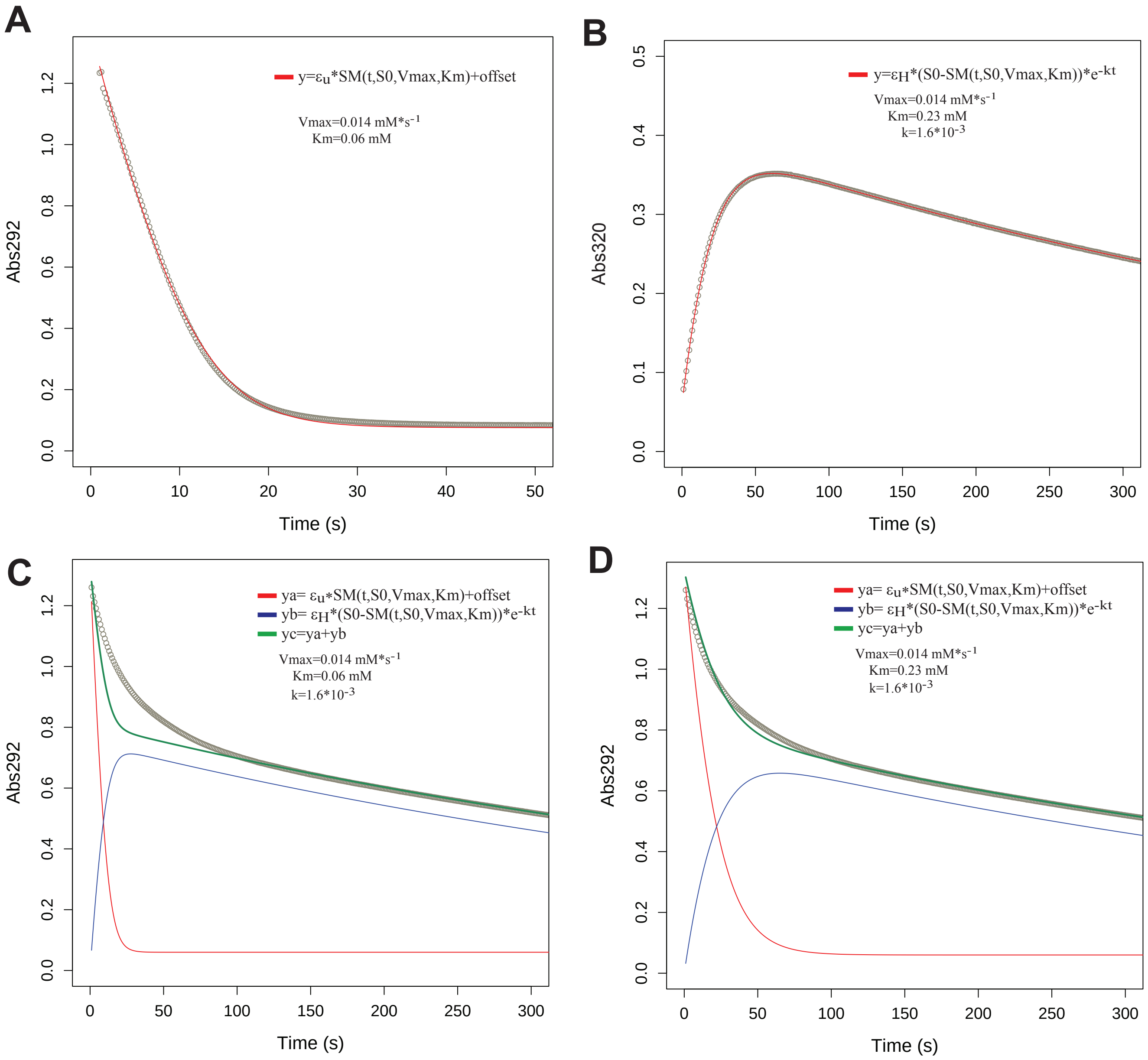

Figure S5. **Fitting of the Uox reaction progression curves**. SM represents the Schnell-Mendoza equation as a function of time (t), initial substrate concentration (S0), maximum velocity (V_{max}), and Michaelis constant (K_m). Substrate and enzymes concentrations are the same as in Fig. 4. (**A**) Variation in absorbance at 292 nm ($\varepsilon 292_{urate}$=12.65 mM^{-1} cm^{-1}) in the presence of Urah fitted with the equation for substrate decrease; the kinetic parameters calculated with the fitting procedure are indicated. (**B**) The variation in absorbance at 320 nm ($\varepsilon 320_{HIU}$=3.5 mM^{-1} cm^{-1}) in the absence of Urah fitted with the equation for enzymatic product formation and non-enzymatic exponential decay (k=1.6 x 10^{-3}). (**C**) The variation in absorbance at 292 nm in the absence of Urah compared with the curve resulting from the combination of urate decrease (red) and HIU formation and decay (blue; $\varepsilon 292_{HIU}$=7 mM^{-1} cm^{-1}) using the kinetic parameters calculated in A. (**D**) The same comparison as in C using the kinetic parameters calculated in B.

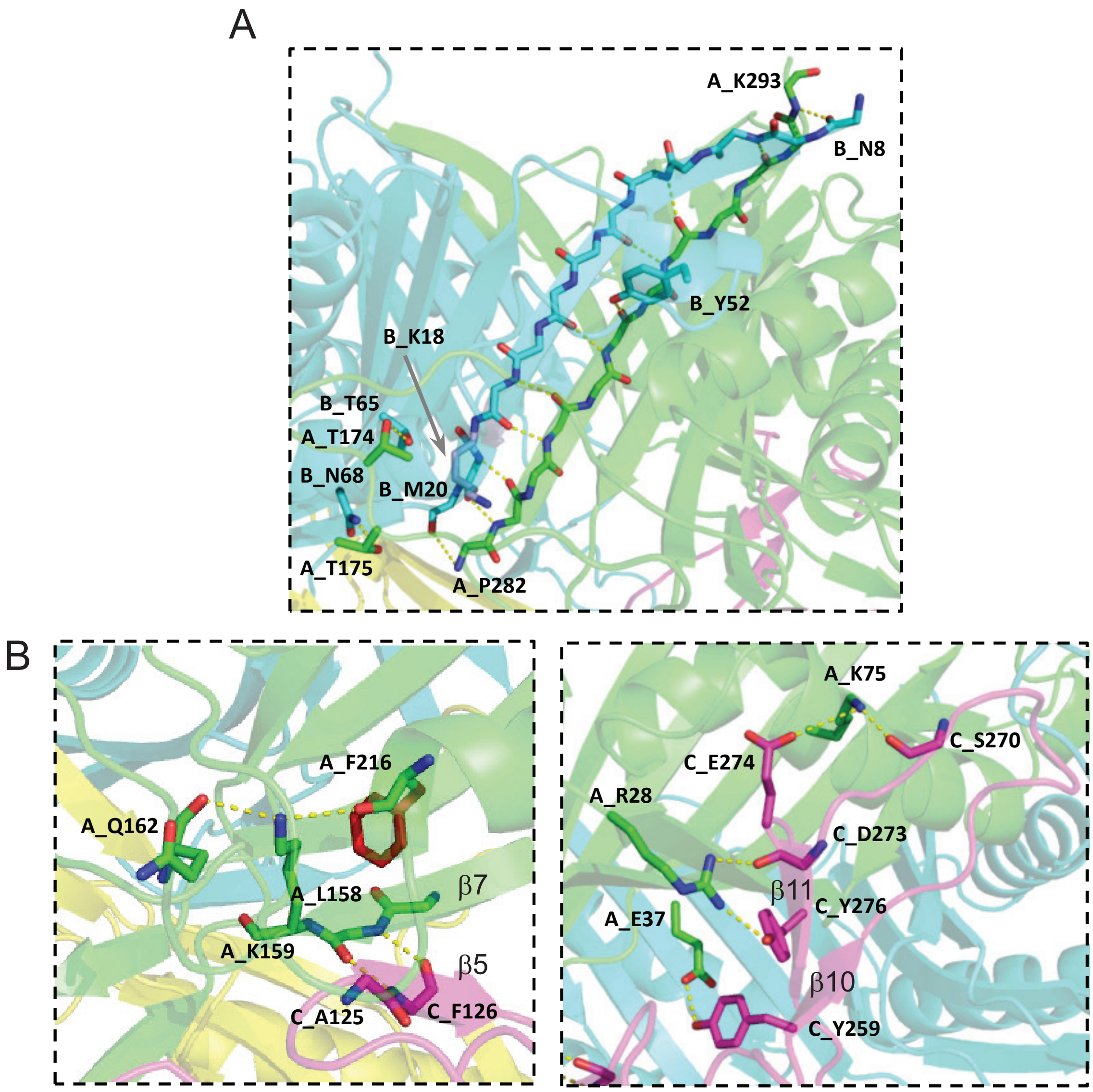

Figure S6. **Dimer and inter-dimer interfaces in *Dr*Uox**. (A) Detail of the main dimer interface between subunit A (green) and B (cyan); residues involved in dimerization interactions and the catalytic Lys18 are represented in sticks. Yellow dotted lines indicate polar interactions (H-bonds and salt bridges). (B) Detail of the two main dimer-dimer interfaces between subunits A (green) and C (magenta). Left panel: dimer-dimer interface involving strands β5 and β7; the side chain of F216 (red stick) is indicated together with the interaction network involving K159 and Q162. Right panel: dimer-dimer interface involving the loop between strands β10 and β11.

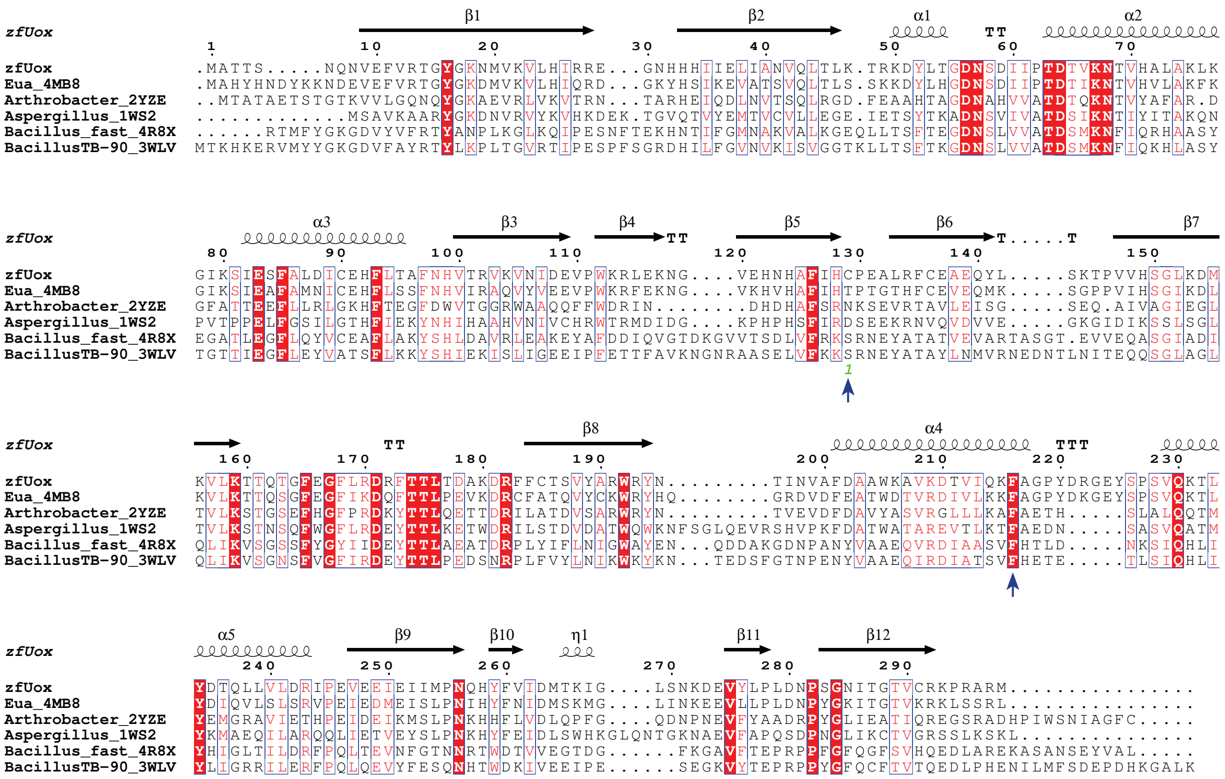

Figure S7. **Multiple alignment of Uox with known structure.** Sequences are identified by the organism name (Eua=euarchontoglires ancestor) followed by the PDB code. Identical residues are shaded in red. Secondary structure elements derived from the zfUox atomic coordinates are shown over the alignment. The cysteine residue (C129) involved in inter-subunit disulfide bond and the phenylalanine residue (F216) substituted by side-directed mutagenesis are indicated.

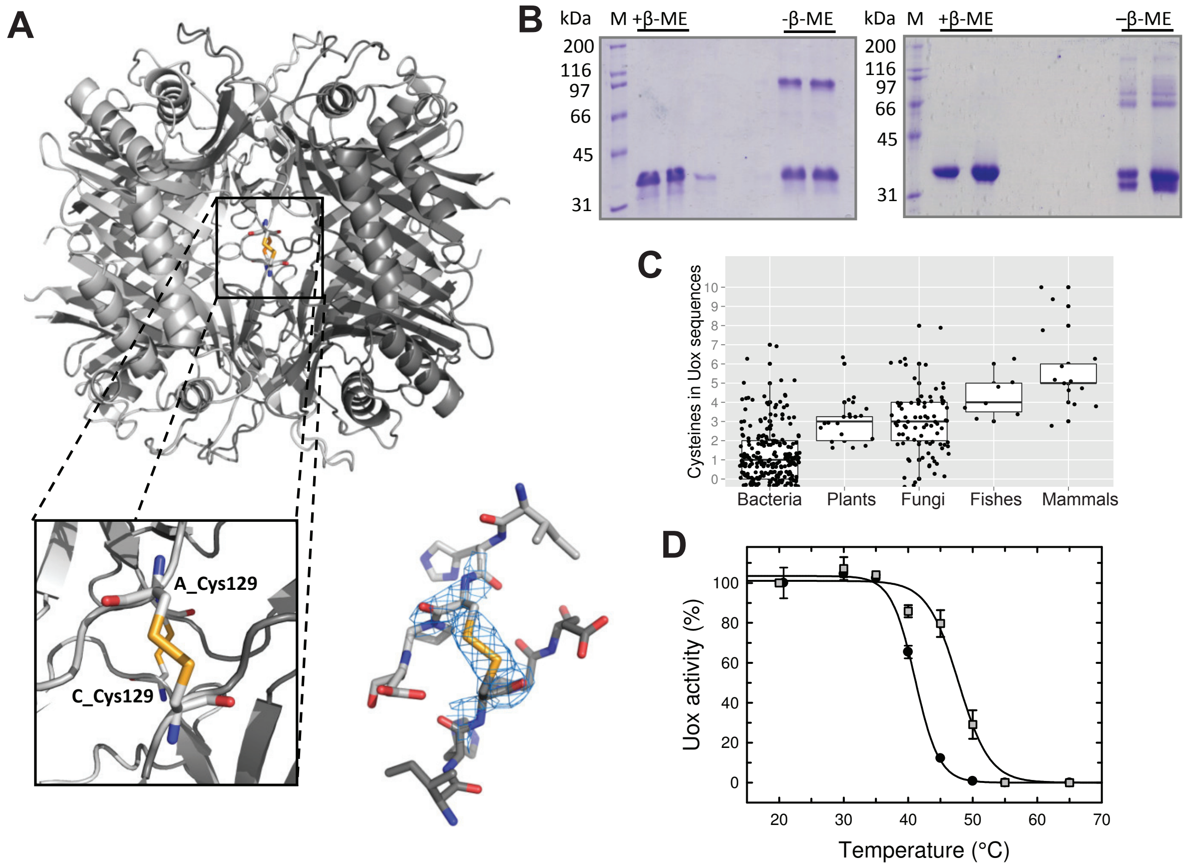

Figure S8. ***Dr*Uox disulfide bonds in the crystal and in solution**. (**A**) Cartoon view of the *Dr*Uox tetramer with magnification of the disulfide bond (in sticks) at the tetramer interface; the electron density map for Cys129 is shown at 1.83σ contour level. The distance between sulphur atoms of cysteine residues of different subunit is 2.1 Å. (**B**) SDS-PAGE analysis of S-alkylated (left panel) or non-alkylated (right panel) *Dr*Uox in reducing (+β-Me) or non-reducing (-β-Me) conditions. In the absence of the reducing agent, both the monomer and dimer of non-alkylated *Dr*Uox appear as split bands, likely due to the formation of non-native disulfide bonds. (**C**) Median (thick lines), first and third quartile (thin lines) of the distribution of the number of cysteines in individual Uox sequences (black circles) grouped according to taxonomy. (**D**) Residual Uox activity after 30 min incubation at various temperatures in reducing (squares) or non-reducing (circles) conditions; error bars are standard deviations between three independent replicates; data were fitted to a three-parameter sigmoid function for eye guidance.
